# Volatility spillovers across sectors and their magnitude: A sector-based analysis for Australia

**DOI:** 10.1371/journal.pone.0286528

**Published:** 2023-06-01

**Authors:** Duc Hong Vo

**Affiliations:** Research Centre in Business, Economics & Resources, Ho Chi Minh City Open University, Ho Chi Minh City, Vietnam; Universiti Malaysia Sabah, MALAYSIA

## Abstract

While spillover across equity markets has been extensively investigated, volatility spillover across sectors has largely been under-examined in the current literature. This paper estimates the sectoral volatility using the ARMA-GARCH model and its spillover across Australian sectors on the VAR framework during the 2010–2021 period. We then identify breakpoints in market volatility during the Covid-19 pandemic using a wavelet methodology. We find that volatility spillover across Australian sectors is very significant at 60 per cent from 2010 to 2019, reaching 90 per cent during the Covid-19 pandemic in 2020. The spillover then reverts to its pre-pandemic level in 2021. *Consumer Staples* and *Industrials* are the significant risk transmitters, whereas *Financials* and *Real estates* are the most significant risk absorbers. Our findings also indicate that *Real Estate*, *Health Care*, and *Financials* record the most significant increase in volatility of more than 300 per cent. Policy implications regarding risk management across Australian sectors have emerged, particularly during extreme events such as the pandemic.

## 1. Introduction

The Australian economy is ranked 13th globally using nominal GDP and 18th using the PPP-adjusted GDP in 2020 (World Bank, 2021). These GDP rankings are undoubtedly due to the contributions of various economic sectors in Australia. Over the 2010–2020 period, the services sector is a major contributor to the Australian GDP with an average share of approximately 66 per cent, followed by industry and agriculture with an average GDP share of approximately 24 per cent and 2 per cent, respectively. Furthermore, the contribution of these sectors to the Australian economy has remained stable for the past ten years, further highlighting the importance of the services sector in the Australian economy.

**Fig 1:**
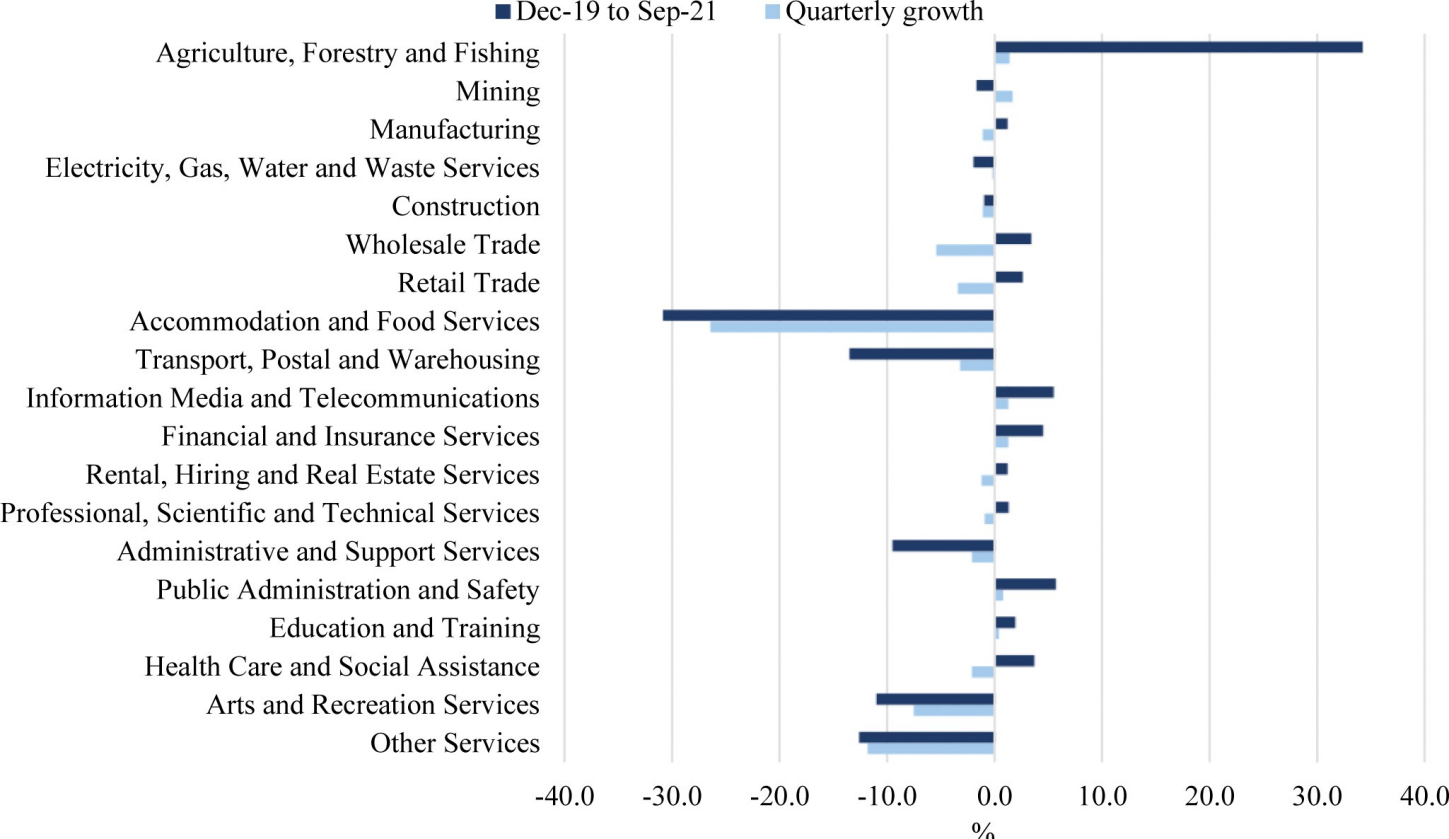
Gross value added by the Australian industries during the Covid-19 pandemic. Source: ABS, Australian National Accounts: National Income, Expenditure and Product September 2021.

However, such stability was interrupted by the emergence of the Covid-19 pandemic, causing the Australian economy into a recession for the first time in nearly three decades in September 2020. The country’s GDP dropped by 7 per cent in the June 2020 quarter, which became the worst fall ever to the Australian economy (The Australian Bureau of Statistics, 2021). The decline in Australia’s GDP results from a significant decrease made by the services-related industries within the services sector. Fig 1 presents the gross value added (GVA) by Australian industries during the Covid-19 period from December 2019 to September 2021. The quarterly growth covers the third quarter of 2021, from July 2021 to September 2021. Almost all industries generally have low or negative percentage growth in GVA, except for agriculture, forestry, and fishing. The services-related industries significantly decline in the GVA. Hospitality, tourism, and other service-related sectors recorded the most significant reductions. The transport, postal and warehousing industries also witnessed a considerable decline in GVA.

**Fig 2:**
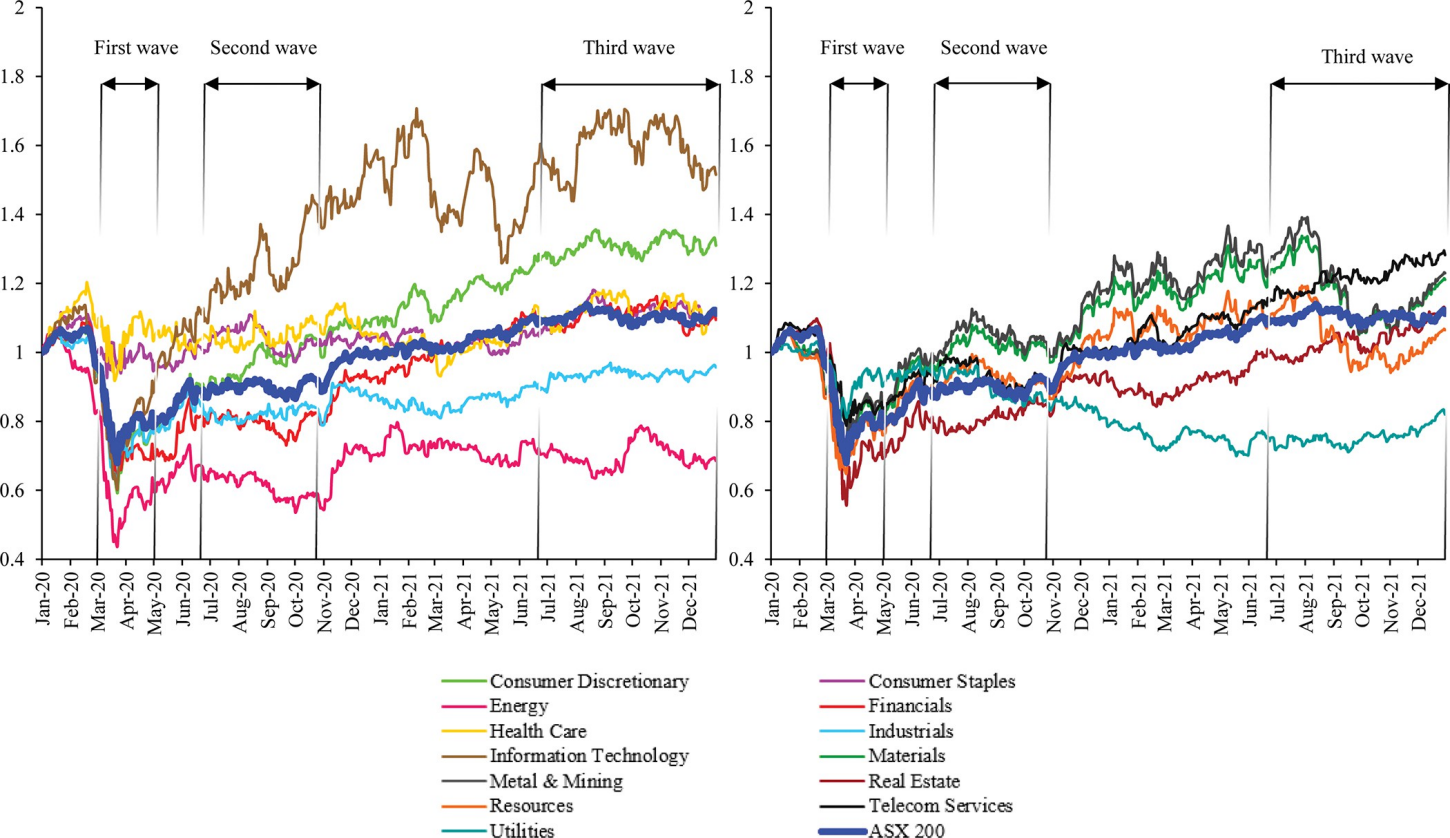
The volatility of Australia’s stock market index and 13 Australian sector indices, January 2020 –December 2021.

The downturn of various industries is usually linked with significant volatility in the financial market. Specifically, Australia’s stock market index (ASX200) and selected sector indices have significantly fluctuated throughout the three Covid-19 waves in Australia, as illustrated in Fig 2. Overall, the volatility was most evident during the first Covid-19 wave in April 2020, when there was a rapid plunge in the price of the ASX200 and all other sector indices simultaneously. From March to April 2020, the ASX200 was down 39 per cent from its previous peak. Among all Australian sectors, the energy sector experienced the largest drop of 62 per cent, while the consumer staples sector exhibited the smallest reduction of 19 per cent. These free falls in the Australian stock market were attributed to the nationwide lockdown during the first Covid-19 wave in Australia.

Later, the Australian stock market exhibited signs of recovery in the second and third Covid-19 waves. The second wave occurred from June to late October 2020 as Victoria entered a four-month strict lockdown due to the virus outbreak in Melbourne. Meanwhile, the third wave lasted from June 2021 to the end of the year, witnessing the occurrence of the Delta variant in June 2021 and the Omicron variant in early December 2021. The outbreak of the Delta variant had left nearly half of Australia’s population and major cities in lockdown until the vaccination covered 90 per cent of the population in December 2021. Since then, the Australian government has gradually lifted the lockdown despite the occurrence of the Omicron variant in early December 2021. The end of the lockdown in the latter two waves has caused the volatility, though still significant, to be much less volatile than in the first wave. Notably, the Omicron variant appears to have little effect on the volatility because Australia has effectively opened since November 2021.

Fig 2 presents the volatility in the Australian stock market and market indices for 13 Australian sectors. The sectors in an economy possess a connectedness in which strong volatility occurring in one sector may also impact other sectors’ volatility [1, 2]. This mechanism is generally known as volatility spillover, a typical characteristic of financial crises and economic downturns [3, 4]. Thus, the Covid-19 pandemic in Australia might have contained volatility spillover among Australian sectors. Examining the volatility spillover across the Australian sectors during extreme events such as the Covid-19 pandemic is important in shaping public policies and investment decisions. This important issue of volatility spillover across sectors during the Covid-19 pandemic appears to be largely ignored in the existing literature. Previous studies have addressed the sectoral volatility spillover within certain financial markets other than Australia during the Covid-19 pandemic, such as China [5, 6], the US [3], Europe [7], and the G7 countries [1].

Unlike previous studies, this study is conducted to achieve the following research objectives. First, we estimate the market volatility across 13 Australian sectors during 2010–2021 and identify the most vulnerable sectors to previous shocks using the ARMA-GARCH model. We then investigate the market volatility spillover across these sectors for the entire research period and then compare it between the pre-Covid-19 pandemic and the during-Covid-19 pandemic. This analysis allows us to identify the sectors considered risk transmitters or receivers among Australian sectors. We use the VAR framework for this analysis. Using the wavelet methodology, our study is then extended to determine the breakpoints in market volatility across the Australian sectors during the Covid-19 pandemic.

The contributions of this study are threefold. *First*, the market volatility across sectors has been under-examined, especially during the Covid-19 pandemic. A paper by Choi et al. [8] focuses on the impacts of the global financial crisis on volatility across 11 Australian sectors. Using different estimation techniques, our paper focuses on the effects of the Covid-19 pandemic across 13 Australian sectors. Two Australian sectors, including Metals & Mining and Resources, are excluded from Choi et al. [8] analysis. However, these sectors are included in our analysis because of their significant contribution to the Australian economy. Also, we focus on the impacts of the Covid-19 pandemic on volatility, especially at the “breakpoints” in market volatility, which have attracted significant attention from policymakers, practitioners, and academics in the past 24 months. *Second*, previous studies have examined the volatilities across equity markets and their connectedness. However, these studies have ignored the volatility patterns and their transmission across sectors. Using the wavelets transform methodology. Our study estimates the magnitude to which market volatility changes across sectors at the breakpoints during the Covid-19 pandemic. The wavelet transform approach, widely applied in the image and signal processing area of research, has hardly been used in business studies, particularly in market volatilities and their spillovers. *Third*, we investigate the change in the risk level for various investment holding periods using the multiresolution analysis.

Following this introduction, the remainder of the paper is structured as follows. Section 2 discusses and synthesizes relevant theories and empirical evidence on the issue. Section 3 presents the research methodology and data. Section 4 discusses empirical results, followed by the concluding remarks and policy implications in section 5 of the paper.

## 2. Literature review

Several studies have examined the market volatility and the spillover effects across markets, especially the effects of the Covid-19 pandemic on the market risk in the past two years. These analyses have employed the Generalized Autoregressive Conditional Heteroskedasticity (ARCH/GARCH) models and the Vector Autoregression (VAR) models [1–4, 6, 8–13].

Laborda and Olmo [3] consider understanding the sectoral volatility transmission crucial since the increase in market volatility could be the lead indicator for financial crises. Also, an increase in market volatility can be considered a reflection of an increase in different asset connectedness [13]. Findings from Zhang et al. [1] confirm that the correlation between global financial markets is gradually strengthening due to economic integration and financial globalization. Recently, the Covid-19 pandemic outbreak has been considered to impact the market volatility and the volatility spillover tremendously. Hasan et al. [11] argue that the stock market is more sensitive to the pandemic outbreak than the real economy. Bissoondoyal-Bheenick et al. [9] confirm that stock return and volatility spillover have significantly increased with the rapid outbreak of the Covid-19 pandemic. These findings align with Aslam et al. [7] and Akinlaso et al. [14]. During the pandemic, the total directional volatility spillovers among twelve European stock markets remain high [7]. In Tunisia, the Covid-19 outbreak has caused the conventional stock market to transmit negative volatility spillovers on the Islamic stock markets [14].

Scholars have compared the volatility spillover during the Covid-19 pandemic and the global financial crisis (GFC). Choi [15] reports that the total volatility spillover among South Korea, Japan, China and the US stock markets is more vigorous than during the GFC. During the pandemic, the South Korean stock market appears to be the main volatility transmitter among the four markets. Also, findings from Gunay and Can [16] indicate that the Covid-19 pandemic causes more volatility spillover than the GFC. Advanced economies especially experience a greater magnitude of volatility spillover compared to emerging markets. In addition, increasing volatility spillover during Covid-19 is also found among financial assets and commodities [10, 17–20].

Ghorbel and Jeribi [10] observe a spike in volatility spillover across the US market indexes, cryptocurrencies, and oil prices during the Covid-19 pandemic. Wen et al. [20] report that the stock market has magnified its volatility spillover on the commodity market in China. Samitas et al. [19] present a peak of total volatility spillovers among fine wine, equities, bonds, crude oil, commodities, gold, copper, shipping and real estate. Farid et al. [18] reveal significant two-way volatility spillovers between precious metals in the US during the Covid-19 period. Elgammal et al. [17] present the relatively significant bidirectional volatility spillovers between global stock markets and both energy and gold markets, as well as cross-volatility spillovers from Energy to gold markets.

Many scholars have examined the stock market’s sectoral volatility and spillover effects. For instance, the volatility connectedness across ten sectors in China’s stock market is examined in Su and Liu’s [6] analysis. The Chinese stock market’s sectoral spillover effect is significantly high. The major risk transmitters are the Consumer Discretionary, Industrials, and Materials sectors. In the US, Choi [21] finds that the Covid-19 emergence increases volatility spillovers across 11 sectors in which Energy, Consumer Discretionary, and Consumer Staples sectors appear to be the major volatility shock transmitters.

In the Australian context, Choi et al. [8] examined the spillover effects across 11 sectors from 1 January 2004 to 31 December 2016. They employ the spillover index methodology of Diebold and Yilmaz [13] and focus their analysis on the global financial crisis (GFC) impacts on Australia’s sectoral volatility connectedness. Their results confirm that the GFC substantially increases the sectoral volatility spillover. Besides, the financial sector is the main volatility transmitter among all Australian sectors.

Our literature review indicates that while spillovers across financial markets and products have been intensively investigated, volatility spillovers across the Australian sectors have been under-examined, particularly under extreme events such as the Covid-19 pandemic. Besides, various techniques should be used to ensure the robustness of the findings, such as the ARMA-GARCH model for estimating volatility, the VAR framework for investigating volatility spillovers and the wavelet methodology for identifying breakpoints in volatility across sectors. These gaps warrant our analysis, as discussed in the following sections, to be conducted.

## 3. Data and research methodologies

### 3.1. Data and sampling

Daily closing prices of thirteen Australian sectors’ indices are used for 12 years, from 2010 to 2021. These sectors include *Consumer Discretionary*, *Consumer Staples*, *Energy*, *Financials*, *Health Care*, *Industrials*, *Information Technology*, *Materials*, *Metals and Mining*, *Real Estate Investment Trust (REIT)*, *Resources*, *Telecom Services*, and *Utilities*. In addition, the sub-period of 2020–2021 is also used to examine the impacts of the Covid-19 pandemic on market volatility across Australian sectors. All data are collected from Thomson Reuters.

### 3.2. Methodologies

#### 3.2.1. The ARMA-GARCH approach to estimating volatility

We employ the ARMA-GARCH model to estimate the market volatility and to determine the volatility patterns across 13 Australian sectors. We note that the GARCH model will likely capture both volatility clustering and heteroskedasticity of the variables. The GARCH mode is generally considered more advantageous than the ARCH model in estimating the residuals with unstable magnitude obtained from the AR or ARMA model [22]. We also employ the ARMA model to analyze the market volatility (${{\hat{\sigma }}^{2}}_{it}$). The market return for each sector is estimated as follows.


$$X_{it}=\ln\left(\frac{D_{it}}{D_{i,t-1}}\right)$$

Where: D_it_ denotes the closing price of the index i at time t, X_it_ represents the change in the price of the index i at time t.

The optimal lag length of AR (*r*) and MA (*s*) are determined using the Bayesian Information Criterion (BIC) maximization.

$$\left(1-\sum_{i=1}^{r}\beta_{i}B^{i}\right)X_{t}=\left(1+\sum_{i=1}^{s}\gamma_{i}B^{i}\right)\varepsilon_{t}$$

where: *B*^*i*^ stands for the backshift operator. *β*_*i*_ and *γ*_*i*_ represent the coefficients of the auto-regressive (AR) and moving average (MA) parts of the ARMA model. *ε*_*t*_ denotes the residual that is expected to be independently and identically distributed.

Next, tests for the heteroskedasticity of the dataset to confirm the ARCH effect are conducted using the Ljung-Box portmanteau (*Q*) test [23].

$$Q=n(n+2)\sum_{j=1}^{m}\frac{1}{n-j}{\hat{\rho }}^{2}(j)\to \chi_{m}^{2}$$

where: *m* stands for the degree of freedom to examine the model stationarity. ${\hat{\rho }}^{2}(j)$ is the estimated autocorrelation of lag *j*. *n* denotes the number of observations.

The null hypothesis of this test is that the series follows a white noise process, implying no ARCH effects. When the null hypothesis is rejected, the return series have ARCH effects. As such, these findings warrant our next step to use the ARMA(*r*,*s*)—GARCH(*p*, *q*) models to extract the conditional variance (market volatility). The GARCH(*p*, *q*) is described as follows:

$${\hat{\sigma }}^{2}=\alpha_{0}+\sum_{i=1}^{q}\beta_{i}\varepsilon_{t-i}+\sum_{i=1}^{p}\gamma_{i}\sigma_{t-i}^{2}$$

#### 3.2.2. The forecast error variance decomposition in a vector autoregression (VAR) model

Our primary objective is to investigate the volatility transmission across various sectors in the Australian stock market. Our next step is to adopt the network analysis approach proposed by Diebold & Yilmaz [24, 25] in our analysis. Under this network analysis approach, the association structure could be identified deeper. The transmission structure’s direction and node weight can also be identified simultaneously [26]. The rich information and simplicity of the interpretation provided by this network analysis method make it a perfect fit for our study’s objectives.

Diebold & Yilmaz [24, 25] construct the spillover index based on the forecast error variance decomposition in a vector autoregression (VAR) model. The procedure of this method is as follows. *First*, the VAR model of order *p* is fitted to the time series of volatility obtained from the ARMA-GARCH process. Our stationarity tests confirm that the 14 series of volatility are stationary at a level. *Second*, using our data up to time *t*, we estimate the forecast of the volatility series for *h* periods ahead and obtain the error variance decomposition of each forecast corresponding to the shocks arising from the same or other network components at time *t*. *Last*, based on the obtained forecast error variance decomposition, we calculate the volatility spillover index of each time series and the total spillover index.

This paper estimates the dynamic volatility spillover effects using a VAR model of order three and the generalized variance decompositions of 12-day-ahead forecast errors with 200-day rolling-sample windows. These parameters are used in the Diebold & Yilmaz [25] study. The optimal order of three in the VAR model is selected based on the final prediction error (FPE) and Akaike’s information criterion (AIC). Furthermore, we also perform the robustness checks using various VAR lags (from lag 1 to lag 5), forecast horizons (5-10-15 days), and rolling-sample window lengths (250-500-750 days).

## 4. Empirical results

### 4.1. Estimating the market volatility using the ARMA-GARCH model

This section presents and discusses the market volatilities for 13 Australian sectors. Table 1 presents the results using the ARMA-GARCH estimation for the entire period. All 13 Australian sectors have both ARCH and GARCH effects. This means that the shocks and volatilities from previous periods affect the markets’ volatility in the current period. Also, the constants for the variance are approximately equal to 0, indicating that the market volatility is substantially affected by shocks and volatility from the previous periods. We now compare the GARCH effects (β) across 13 Australian sectors. We note that the market volatility from *Consumer Staples* is most affected by the volatility from previous periods because of its highest β of 0.948, followed by *Information Technology* (β of 0.94) and *Health Care* (β of 0.913). In contrast, volatility in this period from *Telecom Services* is least affected by volatility from previous periods (the lowest β of 0.774).

**Table 1:** Market volatility across 13 Australian sectors using the ARMA-GARCH model, an entire period of 2010–2021.

Variance Equation	CD	CS	EN	FI	HC	IN	IT	MA	MM	RE	RS	TS	UT
ARCH (α)	0.106***	0.0392***	0.113***	0.119***	0.0562***	0.0804***	0.0441***	0.0825***	0.0778***	0.0897***	0.0935***	0.131***	0.0646***
	(0.0102)	(0.00373)	(0.00715)	(0.00811)	(0.00481)	(0.00720)	(0.00385)	(0.00699)	(0.00702)	(0.00582)	(0.00706)	(0.00791)	(0.00531)
GARCH (β)	0.828***	0.948***	0.884***	0.854***	0.913***	0.880***	0.940***	0.895***	0.905***	0.875***	0.890***	0.774***	0.910***
	(0.0179)	(0.00492)	(0.00797)	(0.00926)	(0.00830)	(0.0131)	(0.00528)	(0.00935)	(0.00850)	(0.0111)	(0.00940)	(0.0142)	(0.00885)
Constant	62***	11.3***	33.4***	35.1***	36**	36.9***	31.7***	42.3***	41.2***	38.5***	39.5***	130***	26.3***
(1e-07)	(9.60)	(2.23)	(5.35)	(4.82)	(5.82)	(6.78)	(4.86)	(8.33)	(8.90)	(7.00)	(8.10)	(12.6)	(5.36)
(α + β)	0.934	0.9872	0.997	0.973	0.9692	0.9604	0.9841	0.9775	0.9828	0.9647	0.9835	0.905	0.9746

*Source*: Data from Thomson Reuters for the period from 2010 to 2021.

*Notes*: Standard errors are in parentheses. Superscripts

*, ** and *** denote the significance at 10, 5 and 1 per cent confidence levels, respectively.

Sectors including **CD**: Consumer Discretionary; **CS**: Consumer Staples; **EN**: Energy; **FI**: Financials; **HC**: Health Care; **IN**: Industrials; **IT**: Information Technology; **MA**: Materials; **MM**: Metals & Mining; **RE**: REIT; **RS**: Resources; **TS**: Telecom Services; **UT**: Utilities.

Besides, the absolute value of (α + β) presented in the last row of Tables 1 and 2 confirms the speed of the mean reversion process for each sector. We note that the higher the absolute value of (α + β) is, the slower the mean reversion of the sector. As such, our findings in Table 1 indicate that all 13 Australian sectors exhibit a slow mean-reversion process and persistent volatility because the sum of the ARCH and GARCH estimated coefficients is approximately close to 1 (α + β < 1). *Energy* has experienced the longest mean reversion (with the highest sum of (α + β) of 0.997). Meanwhile, *Telecom Services* has the fastest mean reversion with the lowest absolute value of (α + β) of 0.905.

**Table 2:** Market volatility across 13 Australian sectors using the ARMA-GARCH model during the Covid-19 period, 2020–2021.

Variance Equation	CD	CS	EN	FI	HC	IN	IT	MA	MM	RE	RS	TS	UT
ARCH (α)	0.314***	0.108***	0.193***	0.176***	0.160***	0.194***	0.232***	0.0995***	0.206***	0.367***	0.107***	0.212***	0.140***
	(0.0700)	(0.0233)	(0.0413)	(0.0238)	(0.0338)	(0.0371)	(0.0656)	(0.0174)	(0.0532)	(0.0581)	(0.0205)	(0.0423)	(0.0207)
GARCH (β)	0.606***	0.829***	0.779***	0.821***	0.786***	0.765***	0.593***	0.841***	0.730***	0.596***	0.835***	0.710***	0.764***
	(0.0752)	(0.0433)	(0.0411)	(0.0156)	(0.0509)	(0.0378)	(0.110)	(0.0236)	(0.0625)	(0.0637)	(0.0289)	(0.0564)	(0.0556)
Constant	17.7***	8.98***	33.1***	5.02***	13.8**	8.97***	82.3***	16.1***	24.4***	18.5***	18.7***	12.3***	14.5**
(1e-06)	(5.55)	(3.48)	(11.6)	(1.75)	(5.44)	(2.42)	(29.6)	(4.41)	(9.32)	(5.34)	(5.64)	(4.03)	(6.15)
(α + β)	0.920	0.937	0.972	0.997	0.946	0.959	0.825	0.9405	0.936	0.963	0.942	0.922	0.904

*Source*: Data from Thomson Reuters for the period from 2010 to 2021.

*Notes*: Standard errors are in parentheses. Superscripts

*, ** and *** denote the significance at 10, 5 and 1 per cent confidence levels, respectively.

We then estimate volatility across sectors using the same ARMA-GARCH approach for the Covid-19 period of 2020 and 2021 in Australia. As presented in Table 2, our results indicate that the ARCH and GARCH effects of all 13 Australian sectors remain significant and persistent (at a 1 per cent significance level). As such, the market volatility is also confirmed to be significantly affected by the shocks and volatility from the previous periods during the pandemic.

**Fig 3:**
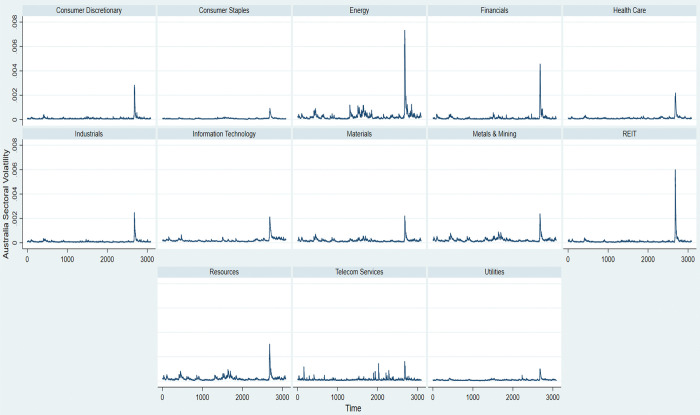
Market volatility across 13 sectors in Australia, entire period 2010–2021.

Fig 3 illustrates the market volatility patterns of all 13 Australian sectors from 2010 to 2021. We focus on these patterns with the focus on the volatility changes across these sectors during the Covid-19 pandemic period of 2020–2021. Over the entire period, *Energy*, *Resources*, and *Telecom Services* are the most volatile sectors in Australia. Meanwhile, the volatility patterns for the remaining 10 Australian sectors are quite stable. However, with the Covid-19 pandemic at the beginning of 2020, major shocks have been observed in all 13 Australian sectors.

Specifically, the volatility patterns across the Australian sectors illustrate the impacts of the Covid-19 pandemic on every Australian. The spike in each sector is observed at around 2,700, which is at the beginning of 2020. Fig 1 presents that *Energy* is the most severely affected sector, which experiences the highest volatility, represented by its variance of approximately 0.75 per cent per day. *REIT* follows with the sector’s volatility hitting its peak at approximately 0.6 per cent during the pandemic. In contrast, *Consumer Staples* and *Utilities* are the most resilient sectors to the Covid-19 pandemic. These sectors record the lowest market volatility during the 2020–2021 pandemic at the volatility’s peak of approximately 0.15 per cent. *Consumer Staples* play an indispensable part in daily life. As such, these sectors are least vulnerable to the pandemic shock. In contrast, *Energy* and *REIT*s are severely affected by quarantine, social distancing, reduction in investment and international trade, and loss in income. As such, these sectors are severely impacted and volatile during the Covid-19 period.

### 4.2. Volatility changes across Australian sectors during the Covid-19 outbreak

After observing the volatility patterns experienced by Australian sectors using the ARMA-GARCH model, we now analyze the changes in the market volatility of all 13 Australian sectors at major breakpoints determined using the wavelet methodology. The wavelet technique, widely used in the image and signal processing area of research, decomposes the entire market returns into smaller components. As a result, many breakpoints are identified during the 2010–2021 period. However, we note that the changes in volatility are not significant between pre- and post-breakpoints compared to the breakpoints identified during the Covid-19 pandemic in 2020 and 2021. As such, this section examines the changes in volatility between the pre-and post-breakpoints during the Covid-19 pandemic across 13 Australian sectors.

Australia recorded the first Covid-19 case on 25 January 2020. To date, three Covid-19 waves in Australia have been generally known. The first and second Covid-19 waves cover the period from 1 March 2020 to 1 May 2020 and from 19 June 2020 to 26 October 2020. The third wave covers the period from 18 June 2021 to December 2021. This period occurred in 2021 with the emergence of the Delta variant and, briefly, the Omicron variant in December 2021. In our analysis, the period from the first Covid-19 case recorded (25 January 2020) until the beginning of the first wave in Australia (29 February 2020) is named pre-Wave 1.

Table 3 presents the results of the volatility changes across 13 Australian sectors using the wavelet methodology. Overall, major identified breakpoints creating substantial changes in each sector’s volatility occurred during the pre-wave 1, wave 1, and wave 2 of the Covid-19 pandemic. The most noticeable breakpoints among 12 Australian sectors, except *Utilities*, occurred during pre-wave 1 of the pandemic. The market volatility of these 12 sectors substantially increases from 63 per cent to 362 per cent between the pre-and post-breakpoints. The first breakpoints across these sectors identified in February 2020 indicate that all sectors suffered from a drastic increase in volatility after the breakpoint, compared to the pre-breakpoint volatility, leading to a substantial increase in the market volatility for each Australian sector.

**Table 3:** Volatility changes between pre-breakpoint and post-breakpoint of 13 Australian sectors using the wavelet approach.

**Breakpoint/ Date**	**CD**	**CS**	**EN**	**FI**	**HC**	**IN**	**IT**	**MA**	**MM**	**RE**	**RS**	**TS**	**UT**
1	pre-Wave 1 (12/02/20)	pre-Wave 1 (24/02/20)	pre-Wave 1 (18/02/20)	pre-Wave 1 (28/02/20)	pre-Wave 1 (13/02/20)	pre-Wave 1 (13/02/20)	pre-Wave 1 (19/02/20)	pre-Wave 1 (24/02/20)	pre-Wave 1 (28/01/20)	pre-Wave 1 (19/02/20)	pre-Wave 1 (28/01/20)	pre-Wave 1 (28/02/20)	
2		Wave 1 (06/03/20)						Wave 1 (30/04/20)	Wave 1 (03/03/20)		Wave 1 (03/03/20)	Wave 1 (03/04/20)	Wave 1 (05/03/20)
3		Wave 1 (30/04/20)							Wave 1 (30/04/20)		Wave 1 (01/05/20)		Wave 1 (03/04/20)
4	Wave 2 (07/07/20)	Wave 2 (06/10/20)	Wave 2 (30/06/20)			Wave 2 (07/07/20)				Wave 2 (06/07/20)	Wave 2 (15/09/20)	Wave 2 (02/09/20)	Wave 2 (06/07/20)
**Breakpoint/ Volatility changes**	**CD**	**CS**	**EN**	**FI**	**HC**	**IN**	**IT**	**MA**	**MM**	**RE**	**RS**	**TS**	**UT**
1	266%	142%	235%	306%	309%	224%	246%	112%	94%	362%	121%	63%	
2		86%						12%	55%		68%	-15%	71%
3		-3%							7%		6%		29%
4	-7%	-21%	13%			2%				-8%	1%	-35%	13%

*Source*: Data from Thomson Reuters for the period from 2010 to 2021.

*Notes*: Pre-Wave 1 covers the period since the first Covid-19 case was recorded in Australia until the beginning of the first Covid-19 wave in Australia (25/01/2020–29/02/2020).

Wave 1 and Wave 2 cover the first (01/03/2020–01/05/2020) and the second (19/06/2020–26/10/2020) Covid-19 waves in Australia.

The date of the breakpoints is reported in parentheses.

Sectors including **CD**: Consumer Discretionary; **CS**: Consumer Staples; **EN**: Energy; **FI**: Financials; **HC**: Health Care; **IN**: Industrials; **IT**: Information Technology; **MA**: Materials; **MM**: Metals & Mining; **RE**: REIT; **RS**: Resources; **TS**: Telecom Services; **UT**: Utilities.

During the pre-wave one period, *REIT*, *Health Care*, and *Financials* record the most significant increase in volatility of more than 300 per cent between the pre-and post-breakpoints. In contrast, *Telecom Services* and *Utilities* experience the lowest volatility increase of approximately 60 per cent between the pre-and post- breakpoint. It is also interesting to note that *Resources* appears to be the first Australian response to the pandemic when its first breakpoint was identified on 28 January 2020, three days after Australia recorded the first Covid-19 case. Besides, *Utilities* appear to be the last sector responding to the pandemic when its first breakpoint was identified in March 2020 during the Covid-19 wave 1 in Australia.

However, the second breakpoint, which is a coincidence with the Covid-19 wave 1, is only identified in six Australian sectors. *Consumer Staples* and *Resources* continue experiencing a significant increase in volatility between the pre-and post-breakpoint of 86 per cent and 68 per cent, respectively. The first breakpoint is identified for *Utilities*, which experiences an increase in volatility of 71 per cent between the pre-and post-breakpoint. We note that four sectors, including *Consumer Staples*, *Metals & Mining*, *Resources*, and *Utilities*, have experienced two breakpoints during the first Covid-19 wave in Australia during March-April 2020 period. However, the change in volatility of these sectors for the second breakpoint is relatively insignificant, except for *Utilities*, with a change in volatility of 29 per cent.

The breakpoints in eight Australian sectors are also identified during the second Covid-19 wave in June-October 2020. However, it is noted that the changes in volatility between the pre-and post-breakpoint are small compared to the first Covid-19 wave. Interestingly, no breakpoint was identified during Covid-19 wave 3 in Australia in 2021. After the first few shocks, the Australian stock market seems familiar with the Covid-19 pandemic. The impacts of Covid-19 on the market volatility are reduced over time, as illustrated in Fig 2.

### 4.3. The spillover effects across Australian sectors during the Covid-19 pandemic

In this section, we examine the spillover effects among 13 Australian sectors over the entire period from 2020 to 2021, employing the vector autoregression (VAR) methodology. All market indices for 13 Australian sectors are tested for stationarity to confirm that employing the VAR model is appropriate. We use the augmented Dickey-Fuller (ADF) test to test the stationarity. All series employed in the model is stationary. Results are available upon request.

Table 4 presents the dynamic connectedness/spillover network between 13 Australian sectors. Each cell in the “To Others” directional row presents the sum of the volatility transmitted to each sector. Meanwhile, the “From Others” column presents the sum of the volatility received from each sector. The “NET" directional connectedness row shows the difference between the volatility transmitted and the volatility received for each sector. The total spillover index (TSI), which is also known as the total connectedness index (TCI), is the number in the lower right-hand corner, which is shaded. The TSI reflects the total average spillover effects across all Australian sectors.

**Table 4:** Spillover effect results of 13 Australian sectors.

	CD	CS	EN	FI	HC	IN	IT	MA	MM	RE	RS	TS	UT	FROM others
Consumer Discretionary (CD)	28.19	13.35	5.11	4.06	7.34	13.27	3.46	3.21	2.98	3.12	3.48	7.04	5.4	71.81
Consumer Staples (CS)	4.13	53.75	3.71	2.38	3.79	4.97	3.5	3.93	3.6	2.7	4.08	6.74	2.73	46.25
Energy (EN)	3.17	10.49	30.49	3.21	5.7	9.55	3.44	7.65	6.82	2.6	9.61	4.36	2.91	69.51
Financials (FI)	4.55	13.49	5.22	28.28	6.28	10.84	5.43	3.8	3.12	3.72	3.4	7.91	3.96	71.72
Health Care (HC)	3.3	10.15	3.63	2.81	44	10	3.12	3.29	2.99	2.31	3.38	6.08	4.95	56
Industrials (IN)	2.81	14.48	4.29	2.92	10.22	38.38	2.82	2.96	2.88	2.67	3.27	7.07	5.22	61.62
Information Technology (IT)	5.25	8.75	4.55	4.54	10.57	6.87	33.27	3.62	2.95	3.13	4.17	8.75	3.57	66.73
Materials (MA)	3.39	6.98	8.27	2.55	4.16	7.91	2.5	21.29	19.92	1.62	15.25	4.12	2.05	78.71
Metals & Mining (MM)	3.31	6.02	7.96	2.18	3.76	7.13	2.31	21.75	22.4	1.6	16.25	3.46	1.87	77.6
REIT (RE)	3.81	10.43	4.32	4.34	4.78	8.88	2.71	3.7	3.58	35.62	4.04	7.4	6.37	64.38
Resources (RS)	3.27	6.06	11.53	1.82	3.21	7.26	2.41	17.21	16.91	1.79	22.94	3.59	2	77.06
Telecom Services (TS)	2.94	6.29	3.51	2.61	3.99	3.7	3.35	2.52	2.34	2.39	2.72	60.36	3.28	39.64
Utilities (UT)	3.42	9.12	4.85	2.71	5.39	6.43	4	2.78	2.85	3.35	3.25	7.13	44.71	55.29
TO others	43.35	115.62	66.96	36.14	69.17	96.81	39.05	76.42	70.94	30.99	72.89	73.66	44.31	**TSI**
NET	-28.46	69.37	-2.55	-35.58	13.17	35.19	-27.68	-2.29	-6.66	-33.39	-4.16	34.02	-10.98	**64.33**

*Source*: Data from Thomson Reuters for the period from 2010 to 2021.

*Notes*: TSI stands for the total spillover index.

Sectors including **CD**: Consumer Discretionary; **CS**: Consumer Staples; **EN**: Energy; **FI**: Financials; **HC**: Health Care; **IN**: Industrials; **IT**: Information Technology; **MA**: Materials; **MM**: Metals & Mining; **RE**: REIT; **RS**: Resources; **TS**: Telecom Services; **UT**: Utilities.

The total spillover index (TSI) of all 13 Australian sectors is approximately 64 per cent during 2010–2021. The TSI result indicates that the spillover effects across sectors are substantial and that the inter-sector connectedness among these sectors is strong. This finding implies that risks from the Australian stock market appear to spread quickly across these 13 sectors.

Based on the net spillover effect results presented in Table 4, we note that *Consumer Staples*, *Industrials*, and *Telecom Services* are the major risk transmitters in the Australian stock market because of their most prominent “NET” values in the volatility transmission network. These figures are 69.37 per cent, 35.19 per cent, and 34.02 per cent, respectively. These sectors are considered the main sources of risk transmission, implying that when significant stock price volatility emerges, the market volatility will spill over to other sectors rapidly. Identifying and considering these characteristics when designing appropriate measures can help policymakers avoid market failure or mitigate the negative impacts on the financial market. In contrast, *Financials*, *REIT*, and *Consumer Discretionary* are respectively the three most significant risk absorbers (with the lowest “NET” values of -35.58 per cent, -33.39 per cent, and -28.46 per cent, respectively).

**Fig 4:**
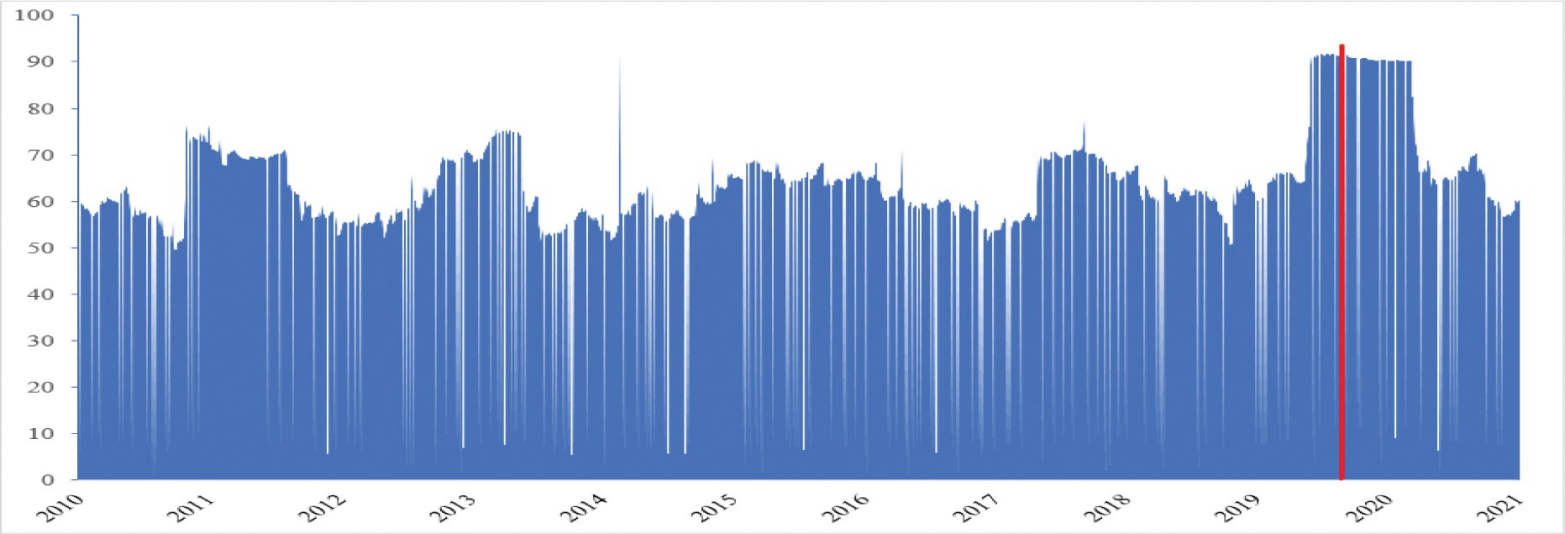
The total spillover effect of all sectors over the 2010–2021 period. *Note*: The red line marks the date of 25 January 2020, when the first Covid-19 cases were recorded in Australia.

Fig 4 illustrates the changes in the trend of the total average spillover index (TSI) over the entire period. The total spillover effects across Australian sectors presented in Fig 2 are consistently high, always more than 50 per cent. However, after the first case of Covid-19 was recorded in Australia on 25 January 2020, the TSI increased over 90 per cent, indicating a substantial market volatility spillover across 13 sectors during the pandemic. When we consider the effects of the Covid-19 pandemic on the market volatility in Australia, we note that high volatility was recorded for all 13 Australian sectors due to the Covid-19 pandemic. As such, the pandemic has caused a substantial increase in the total spillover effects across 13 Australian sectors, supported by the increase of over 90 per cent in the TSI, as presented in Fig 2.

**Fig 5:**
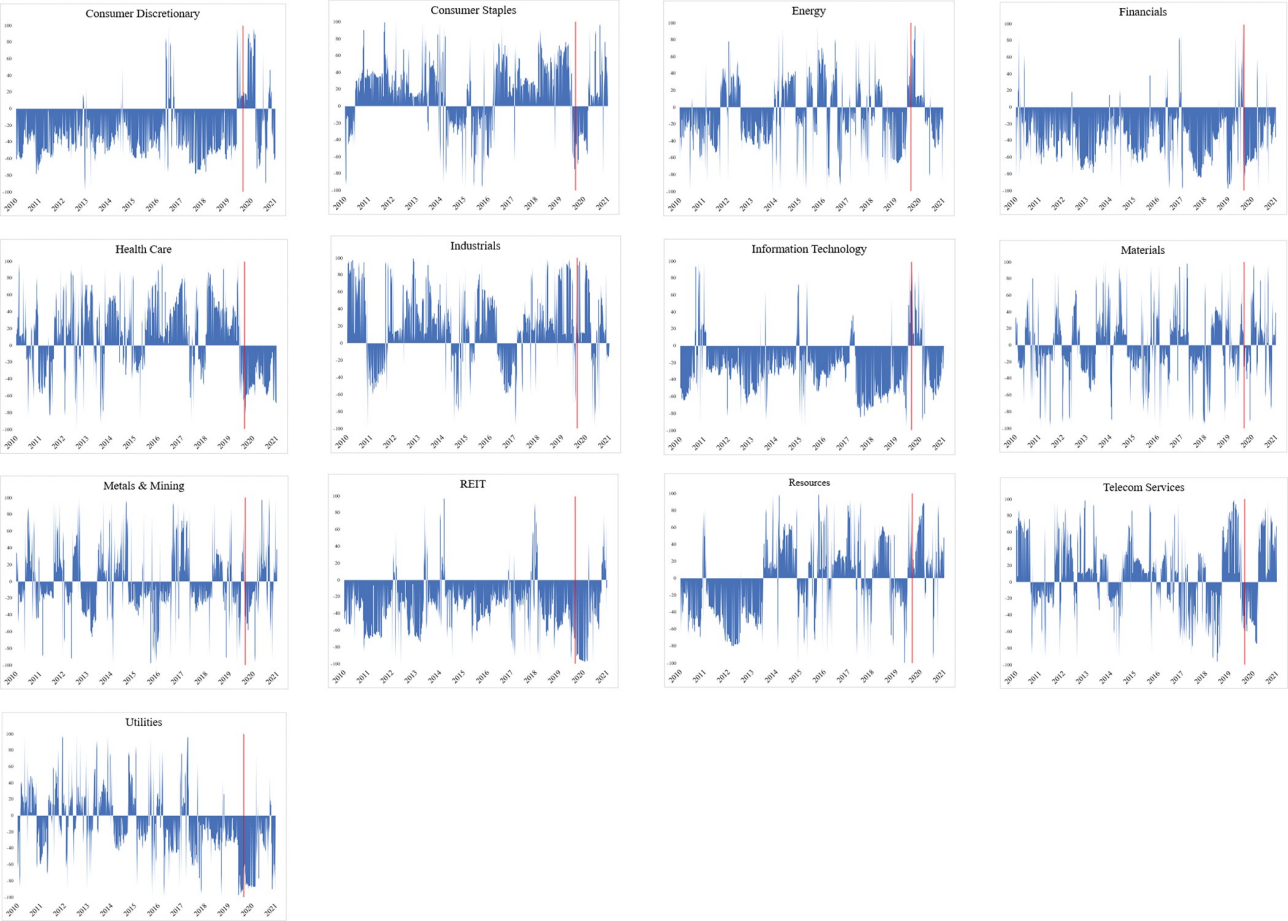
Spillover effects of 13 sectors in Australia over the 2010–2021 period. *Note*: The red line marked the date of 25 January 2020, when the first Covid-19 cases were recorded in Australia.

Fig 5 illustrates the specific changes in spillover effects over the entire period of each of the 13 Australian sectors. In addition, we use the vertical red line to indicate the change in spillover when the first Covid-19 case was recorded in Australia on 25 January 2020 to examine the spillover effects’ differences between pre-pandemic and during the pandemic. Our results from Fig 5 indicate that *Consumer Discretionary*, and *Financials* are the significant risk absorbers during the entire period. In contrast, *Consumer Staples* and *Industrials* are the main risk transmitter over the research period with consistently positive spillover effects.

**Fig 6:**
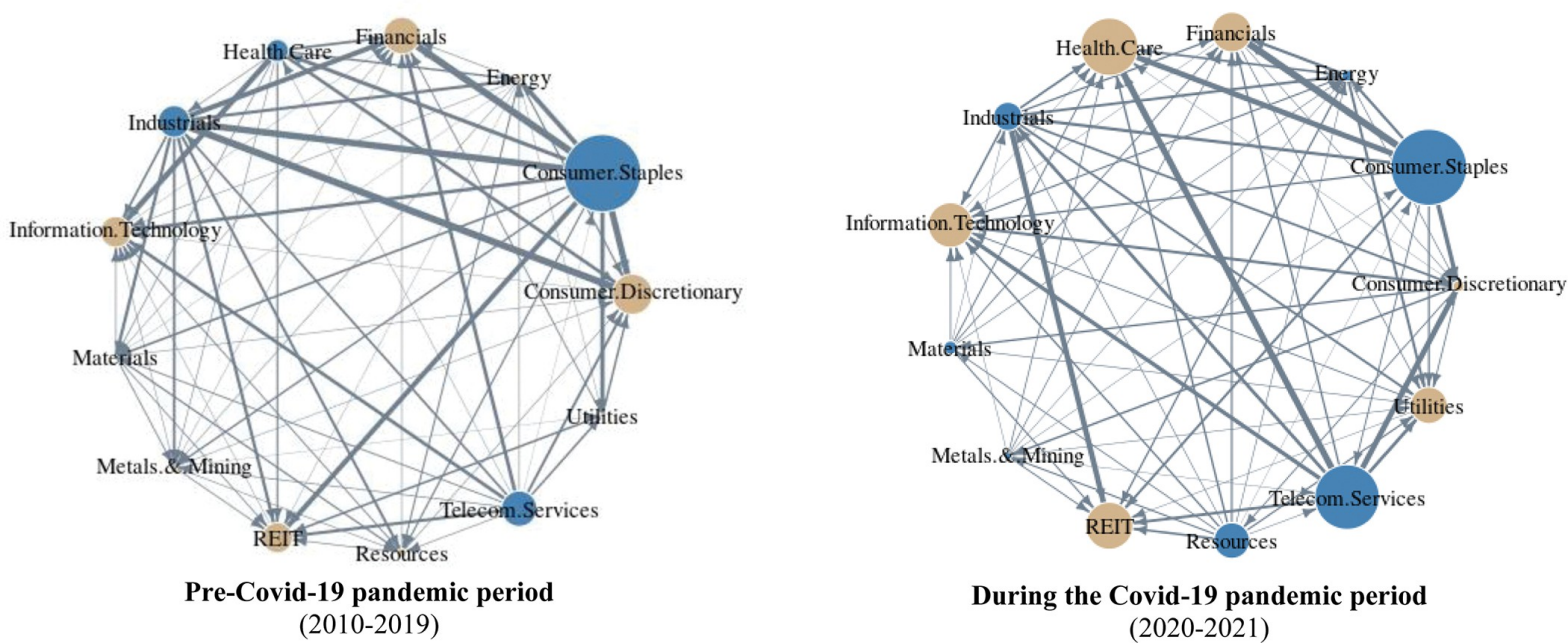
The comparison of the Australian sectoral volatility spillover between the pre-and during the Covid-19 pandemic. *Notes*: Blue (yellow) nodes illustrate the net transmitter (receiver) of shocks. Vertices are weighted by the averaged net pairwise directional connectedness measures. The size of the nodes represents the weighted average net total directional connectedness.

Fig 6 presents the changes and compares the role of 13 Australian sectors in their spillover network before and during the Covid-19 pandemic. *Consumer Staples* is always the risk transmitter because it is the most resilient sector during the pandemic. *Health Care* switches from the risk transmitter to the largest risk absorber after the emergence of the pandemic. *Utilities* changes from a relatively neutral sector to a risk absorber during the Covid-19 period.

### 4.4. Differences in volatility among various investment horizons across Australian sectors during the Covid-19 pandemic

We now shift our attention to the effects of different investment holding periods (investment horizons) on volatility across 13 sectors in Australia using the wavelet methodology. The changes in each sector variances among several holding periods indicate the market risk in terms of volatility that investors must bear when holding the stocks of the relevant sector. We have achieved consistent findings across 13 Australian sectors concerning the relationship between volatility and investment holding periods.

In this section, we select six Australian sectors as illustrations. The first two sectors are *Consumer Staples* and *Utilities*, the two most resilient sectors to the Covid-19 pandemic, as analyzed in the ARMA-GARCH estimation. Also, *Consumer Staples* is the most significant risk transmitter in the spillover network of 13 Australian sectors. The next three selected sectors are *REIT*, *Financials*, and *Health Care*, the most severely affected sectors by the pandemic that record the largest increase in volatility of more than 300 per cent, as presented in Table 3. Additionally, *REIT* and *Financials* are the two largest risk absorbers in the spillover network, while *Health Care* switches from the risk transmitter to the largest risk absorber during the Covid-19 period. The final selected sector is *Energy*, the most volatile sector over the entire period and has the highest volatility spike during the Covid-19 period, as illustrated in Fig 3.

**Fig 7:**
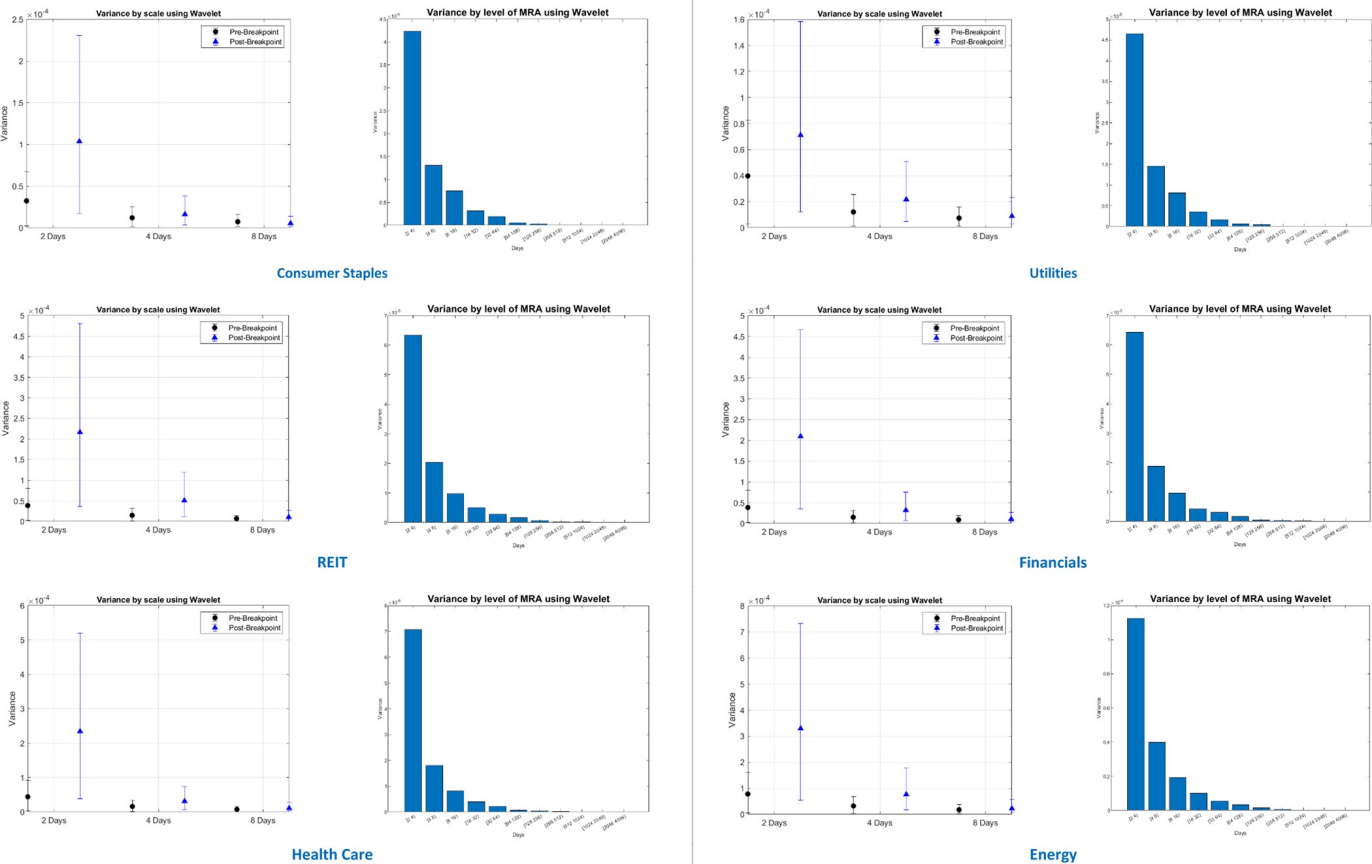
The comparison of changes in variances across various investment horizons for six selected Australian sectors.

Fig 7 illustrates the changes in volatility/variance of different holding periods for these selected sectors. We consider different investment holding periods of 2, 4, 8, 16, and 32 days. The variance changes by scale illustrate the increase in the stock’s variance of each sector’s 2-day, 4-day, and 8-day holding periods between pre-and post Covid-19 breakpoints identified in Table 3. The variance changes by the multiresolution analysis (MRA) level presents the variance of each sector’s stocks of different investment horizons of 2-to-4, 4-to-8, 8-to-16 days, and the like.

Our empirical results indicate that the longer the investment horizon investors hold, the lower the risk in terms of market volatility that the investors must bear. Especially the investment horizons from 2 to 8 days witness the most significant volatility. These findings imply that the short-term investment holding period of 2 days experiences the most extreme risk across the Australian sectors during the Covid-19 pandemic in Australia.

Specifically, our result from Fig 7 shows that changes in variance by the scale of the 2-day holding period from the most resilient sectors, *Consumer Staples* and *Utilities*, are approximately doubled between pre- and post-breakpoints caused by the Covid-19 pandemic. Meanwhile, the variances increased more than four times in the three highest volatility recorded sectors, including *REIT*, *Financials*, and *Health Care*. The largest variance recorded in these sectors is approximately 0.0005 per day. The variance changes by the level also confirm that the holding period from 2-to-4 days exhibits the largest price volatility, which is three times larger in variances compared to the next holding period of 4-to-8 days. *Energy*, the most volatile sector over the entire period, recorded the most significant variance for the 2-to-4 days holding period of 0.00012 per day.

## 5. Concluding remarks and policy implications

Market volatility and its spillover across equity markets have been extensively investigated. However, market volatility spillovers across various Australian sectors have largely been ignored in the literature. Australia is a small open economy where the economic performance of the sectors and the entire economy is significantly volatile in a major event such as the Covid-19 pandemic. As such, this research estimates the market volatility across 13 Australian sectors from 2010 to 2021 to identify the most vulnerable sectors using the ARMA-GARCH model. The market volatility spillovers across the Australian sectors are then examined using the VAR framework for the pre-Covid-19 pandemic and the during-Covid-19 pandemic. Using the wavelet methodology, we also determine the breakpoints in market volatility across the Australian sectors to understand how significant risks have been changed during the Covid-19 pandemic. Key findings from our analysis can be summarized as follows.

First, our empirical analysis from the ARMA-GARCH model indicates that *Consumer Staples* are the most affected sector by the volatility from previous periods. *Information Technology* and *Health Care* are also significantly affected. In contrast, our results indicate that *Telecom Services* are the least affected sector. *Energy* exhibits the longest mean reversion, whereas *Telecom Services* exhibits the fastest.

Second, the total spillover index, indicating the inter-sector connectedness among these 13 Australian sectors, varies between 60 per cent and 70 per cent during the pre-Covid-19 period, from 2010 to 2019. This volatility spillover level indicates that risks from the Australian stock market appear to spread quickly across sectors. The volatility spillover increased to 90 per cent during 2020 when the Covid-19 pandemic emerged. Australia recorded the first Covid-19 case on 25 January 2020. However, the volatility spillover reverted to the pre-pandemic level in 2021 when Australia opened the entire economy without restrictions by the end of 2021. Our analysis also confirms that, among all 13 Australian sectors, *Consumer Staples*, *Industrials*, and *Telecom Services* are the key risk transmitters. In contrast, *Financials*, *REIT*, and *Consumer Discretionary* are the most significant risk receivers.

Third, our wavelet methodology analysis identifies various breakpoints during the Covid-19 pandemic. Three Covid-19 waves have been identified in Australia. The first and second waves cover the March-April 2020 (Wave 1) and June-October 2020 (Wave 2) periods, respectively. The third wave covers July to December 2021 (Wave 3). Market volatility breakdown are identified in 12 Australian sectors for the pre-Wave 1 covering January-February 2020. However, no breakpoint is identified in wave 3. We find that *REIT*, *Health Care*, and *Financials* record the most significant increase in volatility of more than 300 per cent between the pre-and post- breakpoints during pre-wave 1. *Resources* were the first Australian sector to respond to the pandemic when its first breakpoint was identified on 28 January 2020, three days after Australia recorded the first Covid-19 case. Besides, *Utilities* appear to be the last sector to respond to the pandemic.

Fourth, our analysis finds that the longer the investment horizon investors hold, the lower the risk in terms of market volatility. Holding investments for only two days or four days shows the largest volatility. These findings imply that the short-term investment holding period of 2 days experiences the most extreme risk across the Australian sectors during the Covid-19 pandemic in Australia.

Policy implications have emerged based on the results of our study. The services sector, the most significant contributor to the Australian economy, has been hit hardest among all other Australian sectors. The Australian economy is small and open. As such, external shocks can easily and quickly spread to the economy. Given the Australian economy’s size, albeit ranked 13th globally, Australian sectors are significantly vulnerable to external shocks. The services sector then becomes the target for these external shocks. As such, the Australian economy will need to consider diversification strategies to limit the risks from external shocks to the economy. In addition, our empirical results identify Consumer Staples, Industrials, and Telecom Services as the main risk transmitters and Financials, REIT, and Consumer Discretionary as the most significant risk receivers. Therefore, portfolio managers and investors may need to consider diversification of their investment portfolios when these sectors are considered. Once again, the Australian sectors, particularly REIT, Health Care, and Financials sectors, have experienced very significant volatility in response to an extreme event such as the Covid-19 pandemic. Investors may take notes for their investment strategies.

Our study exhibits limitations. The clear mechanism of volatility spillovers across Australian sectors should be established to highlight the most significant factors causing these spillovers. Future studies may focus on a particular industry to understand how risk can transmit or receive from other sectors and to identify the sector as the starting point for volatility spillovers to star. Findings from such studies will provide immense implications for managers and practitioners in understating the nature of risks in the Australian financial market.
